# Deterioration of binocular vision after alcohol intake influences driving performance

**DOI:** 10.1038/s41598-021-88435-w

**Published:** 2021-04-26

**Authors:** Francesco Martino, José Juan Castro-Torres, Miriam Casares-López, Sonia Ortiz-Peregrina, Carolina Ortiz, Rosario G. Anera

**Affiliations:** Laboratory of Vision Sciences and Applications (LabVisGra), Department of Optics, Facultad de Ciencias, University of Granada, Avenida Fuentenueva s/n, 18071 Granada, Spain

**Keywords:** Public health, Oculomotor system, Visual system, Risk factors, Eye manifestations

## Abstract

In this study, we assessed the influence of moderate alcohol intake on binocular vision, vergence system and simulated driving performance by analyzing the interactions between visual deterioration and driving variables. Thirty young healthy subjects were recruited. For the analysis, we measured: visual function (visual acuity and stereoacuity), phorias and fusional reserves. Also, we checked Sheard’s and Percival’s criteria at near and far. The accommodative convergence/accommodation (AC/A) ratio was calculated and vergence facility was also obtained at near. A driving simulator was used to assess driving performance under natural conditions and after alcohol consumption with a breath alcohol content of 0.40 mg/l. Alcohol intake significantly reduced binocular visual performance and vergence function, except for vertical phorias, horizontal phoria at near and Sheard’s and Percival’s criteria at near. Driving performance parameters also presented a statistically significant deterioration after alcohol consumption. A statistically significant correlation was found between the deterioration in overall visual function and overall driving performance, highlighting the influence of the visual deterioration on the driving performance. Moderate alcohol consumption impairs binocular visual and simulated driving performances, implying a greater safety hazard. In addition, deteriorations in binocular visual function and vergence correlated with simulated driving impairment, which indicates that the deterioration of binocular vision due to alcohol consumption affects driving, thus reducing road safety.

## Introduction

Driving under the influence of alcohol represents a serious and significant problem for most countries around the world. According to the World Health Organization (WHO) and its Global Information System on Alcohol and Health (GISAH), more than 3 million people died as a result of harmful alcohol use in 2016^1^, which represents 1 in 20 deaths worldwide. Overall, GISAH statistics establish that the harmful use of alcohol causes more than 5% of global disease burden. Alcohol use is a prevalent factor in road traffic deaths. In 2016, a total of 1,350,000 road traffic deaths were reported, of which 370,000 (27.4%) were related to alcohol consumption^1^. Alcohol increases the risk of road accidents and young people represent the population with the greatest risk of being involved in an accident under the effects of alcohol^2–6^. In the United States of America, over 1000 college students die each year in an alcohol-related accident^7^. Forty-five countries (including the USA and the United Kingdom) still establish their legal limit for breath alcohol content (BrAC) at 0.40 mg/l for driving or the equivalent blood alcohol content (BAC)^1^. BrAC and BAC are expressed in milligrams of ethanol per liter of exhaled air (mg/l) and in grams of ethanol per liter of blood, respectively. BAC in g/l is related to BrAC by a ratio of 2:1, in such a way that a BAC of 0.8 g/l (BAC of 0.08%) equals a BrAC of 0.40 mg/l. Driving is a complex task requiring controlled behavior (control over speed, braking, lane position)^8,9^, a high level of concentration and attention, a capacity for quick decisions and reactions, high processing capacities through the central nervous system, and good visual processing^10–13^. Considering this last point, driving involves the application of different visual functions. In most countries, such as Spain, France, United Kingdom, or the USA, one of the main visual requirements to obtain a driver's license is a visual acuity (on the decimal scale) of greater than or equal to 0.5 (binocularly) or 0.6 (monocularly)^14^, except in the USA where the minimum monocular visual acuity is 0.5. However, some works have reported that other visual functions have an important impact on simulated driving, e.g., contrast sensitivity, stereoacuity, visual field, eye movements, and retinal straylight^10,11,15^. It is also well known that alcohol consumption deteriorates visual performance, in turn affecting visual acuity, contrast sensitivity and glare^16,17^, retinal image quality, and night vision performance^18,19^. Furthermore, alcohol inhibits the nervous system, reducing neural processing and transmission^20^ and affecting binocular vision^21^. Normal binocular vision is an important factor to take into account in the visual fusion process^22^, where the two monocular images are combined into one, providing stereoscopic perception and improved visual performance. Visual aspects such as phorias (latent deviation from the visual axes), fusional reserves (positive and negative fusional vergences), and stereopsis are taken into account when assessing the normality of binocular vision. Phorias are defined as misalignments or compensated deviations of visual axes due to the fusion reflex (a response to maintain the images received on the foveas of each retina). A fusional reserve is defined as the amount of reserve in convergence (visual axes toward nasal) and divergence (visual axes toward temporal) compensating for any phorias relative to a specified distance and consequently, allowing the fusion reflex (single image perception) to be maintained and avoiding diplopia (double vision). The fusion reflex requires sufficient fusional reserves to achieve phoria compensations. Without fusion reflex, stereopsis (one of the most advanced functions of the visual system), that is, the visual system’s capacity to perceive depth, is jeopardized resulting in symptomatic binocular vision. In fact, abnormal binocular vision causes binocular symptoms such as diplopia, blurred vision, headache, and asthenopia (ocular fatigue). Different factors can produce an alteration in normal binocular vision. For example, the use of certain substances, such as cannabis, is known to alter binocular depth inversion^23^. Sleep deprivation also alters normal convergence, thus increasing the exophoria in near and far vision^24^. In this regard, alcohol consumption also diminishes these aspects of binocular vision. Brecher et al.^21^ found that alcohol produces diplopia and decreases visual convergence. Hogan et al.^25^ determined at distance and near variation in heterophorias causing a deterioration in binocular vision due to the tonic effect on the sixth cranial nerve. Munsamy et al.^26^ found a deterioration in heterophoria, fusional vergences, and the near point of convergence in young healthy subjects at a BrAC of 0.1% (0.50 mg/l). Our hypothesis is that deterioration in the binocular visual system due to a moderate alcohol intake could influence driving performance, since driving is a complex task involving several visual functions. In this manner, driving performance could correlate with the deterioration of visual performance showing a causal relationship. No previous studies have characterized the deterioration of the overall binocular visual performance and driving ability after alcohol consumption, particularly when considering the legal limit of 0.40 mg/l of alcohol established for driving in most countries.

With this in mind, the aim of the study was to assess the influence of a moderate alcohol content (BrAC) of 0.40 mg/l on binocular visual performance (by means of visual acuity, stereopsis and fusional vergences) in healthy young subjects with normal binocular vision, and investigate how this affects simulated driving performance. It is the first experimental study to analyze the effects of binocular visual performance on driving performance under the influence of a moderate alcohol intake through several binocular visual parameters, including visual acuity, stereopsis, phorias, fusional reserves, AC/A ratio (the amount of convergence affected by the change in the accommodation), Sheard’s and Percival’s criteria (criteria assessing the correct compensation of the phoria and, in consequence, binocular vision comfort), and vergence facility (capacity of the visual system to respond accurately to vergence changes in a limited time).

## Methods

### Subjects and procedure

The study was approved by the Human Research Ethics Committee of the University of Granada (921/CEIH/2019). Prior to participating in the study, all participants signed an informed consent form in accordance with the Helsinki Declaration, including information on the purpose of the study, the methods used, and the amount of alcoholic beverage they would have to drink. A total of 30 healthy subjects (13 females, 17 males) ranging from 21 to 40 years (26.3 ± 4.4) were recruited in this study. They had a mean body mass index (BMI) of 22.5 ± 2.9 kg/m^2^. The inclusion criteria were: best-corrected monocular visual acuity ≥ 1.0 (decimal notation), no ocular diseases or binocular disorders (normal phorias and stereopsis), being a social drinker with a score of 8 or less on the alcohol use disorders identification test (AUDIT)^27–29^, and not presenting driving simulator sickness^30^. In addition, our study cohort comprised a healthy young population with normal binocular vision, as this is the most typical driving situation for most drivers. We choose a group of healthy young subjects because age is an important factor that could influence the results for the binocularity^31^, driving^30,32,33^, and driving simulator sickness^30^. None of the participants were under pharmacological treatment or had pathological conditions that could be affected by alcohol intake. All participants had had a driving license for at least two years, and they drove at least 2000 km/year. The participants’ mean refractive error (spherical equivalent) was -1.11 ± 1.69 D. The mean pupil size in baseline conditions was of 5.6 ± 0.5 mm. We measured the pupil diameter with the Colvard pupilometer (OASIS Medical, Inc. Glendora, California, USA).

Participants took part in two sessions: the first session under natural conditions (baseline) and the second after alcohol consumption (aAC). The two sessions were performed on different days, with an interval of one week between them, to avoid any ocular fatigue and diminish order effects, just as shown by other studies^15,34^. In the alcohol-intake session, the breath alcohol content (BrAC), defined in milligrams of ethanol per liter of exhaled air (mg/l), was measured using the Dräger Alcotest 6810 (Dräger Safety AG& Co. Lubeck, Germany) breath analyzer which provides good reproducibility^35^. Participants consumed a mixed alcohol beverage (67% orange juice and 33% vodka). They had to reach a BrAC of 0.40 mg/l, and the mean BrAC measured for all participants was 0.40 ± 0.03 mg/l. We used an improved version of the Widmark formula^36^ to calculate the alcohol intake required for each participant to reach this BrAC. Each participant consumed the corresponding alcohol dose within a 30–40 min period. During the second session, we measured the BrAC every 20 min after consuming the alcoholic beverage, so we measured the BrAC five times in total, monitoring for a BrAC of about 0.40 mg/l. All the visual tests were randomized in the two experimental sessions (baseline and aAC) to avoid learning effects influencing the results. The subjects were aware they were consuming alcohol in order to test them in a common real-world situation.

### Visual performance

#### Visual acuity and stereopsis

A full eye examination, including objective and subjective refraction using the endpoint criterion of maximum plus for best visual acuity, was conducted monocularly and binocularly at distance (5.5 m) under photopic conditions. In this manner, the best visual acuity at distance (decimal notation) was measured binocularly under photopic lighting conditions. Stereoacuity was also measured using two different tests: the VistaVision monitor stereotest (DMD MedTech, Villarbasse, Torino, Italy) at distance (5.5 m), and the Randot stereotest (Stereo Optical, Inc., Chicago, USA) at near (40 cm), the latter commonly used in clinical practice. Distance stereoacuity was measured using polarized vertical lines displayed on the monitor. For this, a total of 8 disparities from 300 to 10 arc sec were evaluated. For each disparity, five vertical lines were displayed simultaneously along a row on the monitor, one of which showed a disparity to be perceived stereoscopically. For both tests (distance and near), the observer wore polarized glasses and the task was to recognize stimuli perceived stereoscopically.

#### Phorias and fusional vergences

Phoria is defined as the locus (center) of intersection of the lines of sight, measured with respect to the object of regard, in the absence of a fusional vergence response^37^. Under photopic conditions, distance and near-dissociated phorias (horizontal and vertical) were measured with a phoropter using Von Graefe’s method^38^. In addition, positive and negative fusional vergences (blur, break, and recovery points) were also determined at distance (5.5 m) and near (40 cm) using the phoropter’s Risley rotary prism, which provides high reproducibility and accuracy^38–40^. These parameters were reported in prism diopters (∆).

We also used two methods to calculate the AC/A (accommodative convergence/accommodation) ratio: calculated (Eq. 1) and gradient (Eq. 2).

1 $$ {\text{Calculated AC}}/{\text{A}} = \frac{{15 - {\text{distance phoria}} + {\text{near phoria}}}}{2.5} $$

2 $$ {\text{Gradient AC}}/{\text{A }} = { }\left| {\left( {{\text{near phoria with }}{-}1{\text{D}}} \right){ } - {\text{ near phoria}}} \right| $$

The AC/A ratio is determined by the amount of accommodative convergence induced by a change in accommodation. AC/A values are reported in prism diopters per accommodation diopter (∆/D). It is an important and stable factor^37^ when trying to understand the relation between these two values^41^. In addition, Percival’s and Sheard’s criteria were evaluated to ascertain the presence of any binocular vision alterations. Percival’s criterion defines a rule to anticipate visual discomfort in connection with fusion ranges: the point-of-zero demand should fall in the middle third of the total fusional vergence for comfortable binocular vision^42^. According to Sheard’s criterion, the amount of heterophoria should be less than half the opposing fusional convergence in reserve. Sheard’s criterion is a good discriminator for exo deviations, and Percival’s criterion is good for eso deviations^42^. In our study, a negative value for these criteria (measured in prism diopters, ∆) corresponds to the correct compensation of the respective phoria being measured. In contrast, a positive value represents a decompensated phoria.

We also evaluated vergence facility (VF), which assesses the ability of the fusional vergence system to respond to changes in vergence demands over time. VF is defined as the number of cycles per minute (cpm) through which a stimulus can be fused based on alternating base-in (BI) and base-out (BO) prisms^43^. Optimal reproducibility and accuracy was attained with a near VF test (at 40 cm) was used with a flipper prism of 3∆ BI and 12∆ BO^43^. VF was used to detect any binocular vision problems^44^. We repeated the measurement of all vergence parameters three times for each participant in both baseline conditions and after alcohol consumption. The normal range of vergence facility is considered to be between 10 and 15 cpm^44,45^.

#### Visual performance deterioration

Finally, an overall visual deterioration score (OVDS) was obtained by averaging the z-scores of the deterioration (difference between the aAC and baseline values) of the visual variables including visual acuity, stereoacuity, horizontal and vertical phorias, horizontal negative and positive fusional vergences, vertical fusional vergences, and vergence facility. Equal weight was assigned to all the visual variables. We multiplied the z-scores of some variables by -1, so that for all the variables the more positive the score, the greater the visual deterioration and the worse the visual performance. Z-scores have been widely used^34,46–48^ and are a measurement of how many standard deviations an individual value is away from the group mean.

### Simulated driving and driving performance

A driving simulator represents an accurate means of analyzing driving-related parameters^49,50^. Simulated driving was carried out under photopic lighting conditions, which represent the best conditions for driving performance^51,52^. The driving simulator used in this study consists of three high definition 27″ screens (resolution of 1920 × 1080 pixels) with 180° field of view, a car seat, and the Logitech G27 Racing Wheel (Logitech International SA, Lausanne, Switzerland) comprising a steering wheel, gearshift (six speeds and reverse), and three pedals (accelerator, brake, and clutch). Simax Driving Simulator v4.0.8 Beta software (SimaxVirt S.L., Pamplona, Spain) was used for the driving simulations^15^. The driving scenario, with an itinerary of approximately 12.5 km, was performed in daylight and under good weather conditions. It consisted of three main sections simulating different road environments with moderate traffic. The first section was a 4.5 km long dual carriageway with a speed limit of 120 km/h. The second section was a 6 km single carriageway mountain road with a speed limit ranging from 40 to 90 km/h. The third section was a 2 km inner-city circuit with a speed limit of 40–50 km/h. Participants came to the laboratory at least four times in four separate weeks. All participants received at least two training sessions (separated by a one week interval) to help them acclimatize to the driving simulator^53^, but also to minimize learning effects, as shown in other works^15,34^. During the first training session (week 1), participants completed one full lap of the simulated driving scenario (12.5 km). In the second training session (week 2), participants completed another one full lap of the driving scenario (12.5 km) and performed all visual tests once (results from these tests were not used in the analysis). In both the baseline session (week 3) and the aAC session (week 4) participants carried out one full lap of the driving scenario and performed all visual tests. In these sessions, the visual tests and simulated driving scenario were randomly performed across participants (visual tests were performed before or after the simulated driving). Only results from the baseline and aAC sessions were analyzed. All subjects were instructed to drive normally, while respecting traffic laws. The following variables were measured in order to assess driving performance: mean speed and standard deviation (km/h), distance traveled invading the shoulder (DTIS, m), distance traveled invading the opposite lane (DTIOL, m), total distance traveled outside the lane (TDTOL, m), standard deviation of the lateral position (SDLP, m), and reaction time (s). The reaction time was calculated as the interval between the instant the brake lights turned on in the preceding car and the moment the driver pressed the brake pedal. We also recorded the number of collisions, signaling mistakes, and engine stalls. The SDLP is known to be a valid indicator of impaired behavior with and without alcohol^15,47,54,55^. In line with the OVDS calculation, but also with other studies^34,46,47^, an overall driving performance deterioration score (ODPDS) was also calculated, averaging the z-scores of the deterioration of driving variables included in the three sections: mean speed and standard deviation, DTIS, DTIOL, TDTOL, SDLP, reaction time, collisions, signaling mistakes and engine stalls. In this study, the higher the ODPDS values, the greater the driving performance deterioration.

### Statistical procedures

The statistical analysis was performed using SPSS Statistics v.23.0 software (SPSS Inc., Chicago, IL). The normality of the sample was checked using the Kolmogorov–Smirnov test (n = 30). If the data were normally distributed, a t-test for two-sided alternatives was performed to compare each visual and driving variable separately for the two experimental conditions (baseline and aAC). Similarly, the Wilcoxon signed rank test was used in the case of no normal distribution. The Holm-Bonferroni method to control the family-wise error rate was applied. We also noted the degrees of freedom (DF). Finally, a Spearman correlation analysis was used to study the relationship between the visual deterioration (OVDS) and the driving performance deterioration (ODPDS) scores providing the correlation factor rho (ρ) and the corresponding p-values. Differences were considered statistically significant for p-values < 0.05.

## Results

### Visual performance

#### Visual acuity and stereopsis

Table 1 shows the mean values for pupil diameter and binocular vision function parameters measured under natural conditions (baseline) and after alcohol consumption (aAC). Statistically significant deteriorations were found after alcohol consumption for distance visual acuity (Z = − 4.73, DF = 29, p < 0.001), and distance (Z = − 4.61, DF = 29, p ˂ 0.001) and near (Z = − 4.45, DF = 29, p ˂ 0.001) stereoacuity. A significant increase in pupil diameter was also observed (Z = − 5.58, DF = 29, p ˂ 0.001). As a result, there was a decline in binocular vision parameters for visual acuity and stereoacuity after an alcohol intake that achieved a BrAC level of 0.40 mg/l.

**Table 1:** Mean and standard deviations of pupil diameter and binocular vision function parameters (distance visual acuity and stereopsis) measured at baseline and after alcohol consumption (aAC) giving a BrAC of 0.40 mg/l.

	Baseline	aAC	p-value	Impairment (aAC-baseline)
Pupil diameter (mm)	5.6 ± 0.5	6.3 ± 0.6	< 0.001	0.7 ± 0.5
Distance VA (decimal)	1.35 ± 0.15	1.09 ± 0.15	< 0.001	− 0.26 ± 0.14
**Stereoacuity (arc sec)**				
Distance	25 ± 16	118 ± 91	< 0.001	94 ± 86
Near (Randot)	20 ± 6	36 ± 13	< 0.001	16 ± 11

Mean impairment values for each parameter are included (calculated as the mean difference between aAC and baseline conditions).

#### Phorias and fusional vergences

**Figure 1:**
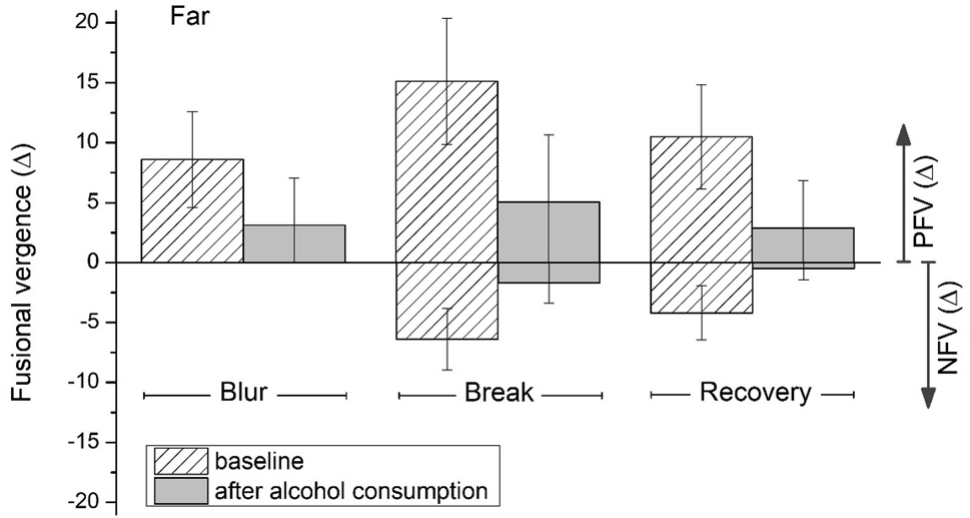
Mean values for the horizontal fusional vergences at distance at baseline and after alcohol consumption (BrAC = 0.40 mg/l). Blur, break, and recovery are shown for the negative (NFV) and positive (PFV) fusional vergences. Error bars show standard deviations.

**Figure 2:**
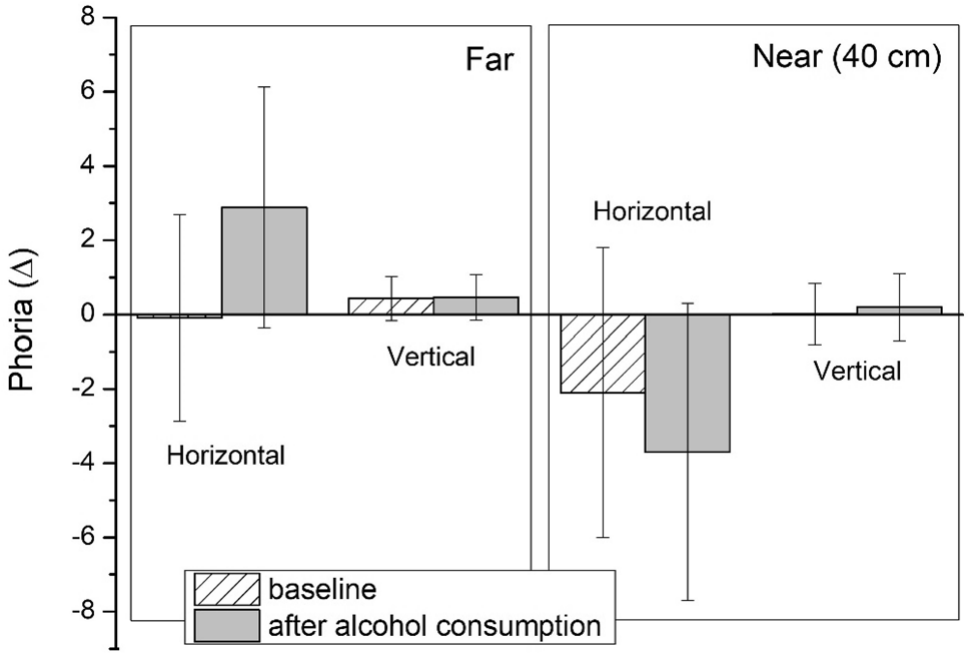
Mean values for distance and near phorias (horizontal and vertical) at baseline and after alcohol consumption (BrAC = 0.40 mg/l). Error bars show standard deviations.

Figure 1 shows the mean values of the distance negative fusional vergences (NFV) and positive fusional vergences (PFV) in the baseline condition and after alcohol consumption (BrAC = 0.40 mg/l), while Fig. 2 depicts distance phorias (horizontal and vertical) at baseline and after alcohol consumption (BrAC = 0.40 mg/l).

According to Table 2 and Figs. 1 and 2, all the distance negative and positive fusional vergences (horizontal and vertical) decreased significantly after consuming alcohol (BrAC = 0.40 mg/l) compared to the baseline session (p ˂ 0.001). All the distance vergence parameters measured at baseline were within normal standard values^56,57^. Moreover, a statistically significant difference was observed and the distance horizontal phoria changed its direction significantly, becoming more esophoric after alcohol consumption (Z = − 4.37, DF = 29, p ˂ 0.001), with a mean value of 2.9 ± 3.2 ∆. In contrast, we did not observe a statistically significant deterioration in the vertical phoria following alcohol consumption (Z = − 0.47, DF = 29, p = 0.638). In addition, Sheard’s and Percival’s criteria were also checked. These criteria assess the correct compensation of the respective phoria measured and the binocular visual discomfort. In Table 2, a negative value for these criteria corresponds to the correct compensation of the respective phoria and, in contrast, a positive value represents a decompensated phoria and binocular visual discomfort. Both criteria employed at distance showed statistically significant deteriorations under the effects of alcohol: Sheard’s criterion, (Z = − 4.66, DF = 29, p ˂ 0.001) and Percival’s criterion, (Z = − 3.52, DF = 29, p ˂ 0.001). Consequently, Sheard’s criterion at distance had a positive mean value after alcohol consumption revealing a decompensated phoria and, therefore, binocular visual discomfort in our subjects.

**Table 2:** Mean values and standard deviations for distance vertical fusional vergences and Percival’s and Sheard’s criteria, measured at baseline and after alcohol consumption (aAC) for a BrAC of 0.40 mg/l.

Far	Baseline	aAC	p-value	Impairment (aAC-baseline)
**Vertical FV (∆)**				
Break	2.2 ± 0.6	0.9 ± 1.0	< 0.001	− 1.2 ± 1.1
Recovery	0.9 ± 0.5	0.1 ± 0.3	< 0.001	− 0.8 ± 0.5
Percival’s criterion (∆)	− 1.4 ± 2.3	− 0.1 ± 1.4	< 0.001	1.3 ± 1.8
Sheard’s criterion (∆)	− 2.4 ± 2.3	1.3 ± 2.5	< 0.001	3.7 ± 2.4

Mean impairment value for each parameter and p-values of the mean comparisons are included.

**Figure 3:**
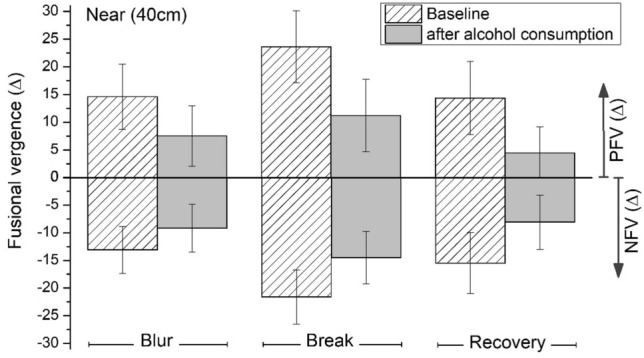
Mean values for the horizontal fusional vergences at near distance at baseline and after alcohol consumption (BrAC = 0.40 mg/l). Blur, break, and recovery are shown for the negative fusional vergences (NFV) and positive fusional vergences (PFV). Error bars show standard deviations.

Figure 2 represents the mean values of the near phorias (horizontal and vertical), while Fig. 3 shows the near negative (NFV) and positive fusional vergences (PFV) under natural conditions and after alcohol consumption.

Similarly, Table 3 and Figs. 2 and 3 show all the parameters of the near phorias and fusional vergences, as well as their criteria and AC/A ratios. All the near vergence parameters measured under natural conditions (baseline) were within normal standard values^56,57^. All the near negative and positive fusional vergence (horizontal and vertical) values decreased significantly after consuming alcohol (BrAC = 0.40 mg/l) compared with baseline (Z = − 4.13, DF = 29, p ˂ 0.001). A decrease of the near horizontal phoria was observed between the baseline condition and after alcohol consumption, but not statistically significant (p = 0.078). Likewise, no statistically significant deterioration was found for near vertical phoria (Z = − 0.69, DF = 29, p = 0.488). At near, no significant changes were reported for the Sheard and Percival’s criteria. Statistically significant deterioration was evident for the vergence facility after consuming alcohol (t(29) = 11.062, p ˂ 0.001) and also for both AC/A ratios: for calculated AC/A ratio (t = 4.47, DF = 29, p ˂ 0.001) and for gradient AC/A ratio (Z = − 3.71, DF = 29, p ˂ 0.001). As a result, the binocular vision was also significantly affected at near.

**Table 3:** Mean values and standard deviations for near vertical fusional vergences, vergence facility, Percival’s and Sheard’s criteria, and AC/A ratio measured at baseline and after alcohol consumption (aAC, BrAC = 0.40 mg/l).

Near	Baseline	aAC	p-value	Impairment (aAC-baseline)
**Vertical FV (∆)**				
Break	3.6 ± 1.3	2.3 ± 1.0	< 0.001	− 1.3 ± 1.1
Recovery	2.0 ± 1.0	0.4 ± 0.5	< 0.001	− 1.6 ± 0.8
Vergence facility (cpm)	14.1 ± 2.8	8.6 ± 3.4	< 0.001	− 5.5 ± 2.7
Percival’s criterion (∆)	− 3.8 ± 2.6	− 3.6 ± 2.9	0.655	0.2 ± 2.3
Sheard’s criterion (∆)	− 6.2 ± 3.5	− 4.7 ± 2.9	0.078	1.5 ± 3.7
Calculated AC/A ratio (∆/D)	5.3 ± 1.4	3.6 ± 1.5	< 0.001	− 1.7 ± 2.1
Gradient AC/A ratio (∆/D)	4.1 ± 1.9	2.3 ± 1.0	0.007	− 1.8 ± 2.2

Mean impairment values for each parameter and p-values of the mean comparisons are included.

### Simulated driving performance

Table 4 shows all the parameters measured at baseline and after alcohol consumption (aAC, BrAC = 0.40 mg/l) that characterize driving performance. All the parameters analyzed in the three sections (dual carriageway, two-lane mountain road and inner city) deteriorated significantly (p ˂  0.05) except signaling mistakes (p = 0.078). Mean speed in the three sections was higher with BrAC = 0.40 mg/l compared to the alcohol-free baseline session. After consuming alcohol, the mean speed in the dual carriageway section was 129.4 ± 13.9 km/h, above the legal speed limit of 120 km/h. Furthermore, the mean speed significantly increased with alcohol intake compared to baseline (t(29) = 4.90, p ˂  0.001). Participants drove significantly more distance invading the opposite lane and also veered more distance invading on the shoulder after consuming alcohol (Z = − 3.94, DF = 29, p ˂  0.001). The SDLP (standard deviation of the lateral position) of simulated driving was significantly higher after alcohol consumption in the dual carriageway (Z = − 4.41, DF = 29, p ˂  0.001) and in the two-lane mountain road, (t(29) = 8.28, p ˂  0.001). Furthermore, a statistically significant increase was also found for the mean reaction times with alcohol consumption (t(29) = 4.83, p ˂  0.001). The number of collisions was higher with alcohol consumption showing a statistically significant deterioration (Z = − 4.55, DF = 29, p ˂  0.001). As a result, simulated driving following alcohol consumption (BrAC = 0.40 mg/l) represented a risk for road safety.

**Table 4:** Mean values and standard deviations for all the simulated driving parameters, measured at baseline and after alcohol consumption (aAC; BrAC = 0.40 mg/l), including the mean change for each parameter, calculated as the averaged difference between aAC and baseline values.

	Baseline	aAC	p-value	Change/impairment (aAC-baseline)
**Dual carriageway**				
MS (km/h)	116.9 ± 4.9	129.4 ± 13.9	< 0.001	12.5 ± 14.0
MSSD (km/h)	8.0 ± 3.0	11.5 ± 4.9	0.012	3.5 ± 5.7
SDLP (m)	0.54 ± 0.12	0.75 ± 0.22	< 0.001	0.21 ± 0.18
DTIS (m)	90.0 ± 119.2	267.8 ± 231.3	< 0.001	177.8 ± 196.4
**Two-lane mountain road**				
MS (km/h)	55.5 ± 1.4	58.4 ± 5.4	0.035	2.8 ± 5.1
MSSD (km/h)	21.4 ± 2.2	23.9 ± 5.0	0.048	2.5 ± 5.2
SDLP (m)	0.55 ± 0.09	0.76 ± 0.16	< 0.001	0.21 ± 0.14
DTIS (m)	43.0 ± 58.6	227.8 ± 257.3	< 0.001	184.8 ± 238.4
DTIOL (m)	309.4 ± 241.5	562.1 ± 418.2	< 0.001	252.7 ± 334.4
TDTOL (m)	352.5 ± 227.9	789.9 ± 478.8	< 0.001	437.5 ± 404.5
RT (s)	0.82 ± 0.13	0.93 ± 0.14	< 0.001	0.11 ± 0.12
**Inner city**				
MS (km/h)	30.5 ± 4.6	36.1 ± 6.4	0.008	5.5 ± 8.1
MSSD (km/h)	16.4 ± 3.4	21.2 ± 5.7	< 0.001	4.7 ± 5.8
**Events**				
Collisions (times)	0.1 ± 0.3	4.3 ± 3.7	< 0.001	4.3 ± 3.6
SM (times)	0.0 ± 0.0	0.3 ± 0.8	0.078	0.3 ± 0.8
ES (times)	1.3 ± 1.3	3.8 ± 4.4	0.009	2.5 ± 4.3

*MS* mean speed, *MSSD* mean speed standard deviation, *SDLP* standard deviation of the lateral position, *DTIS* distance traveled invading the shoulder, *DTIOL* distance traveled invading the opposite lane, *TDTOL* total distance traveled outside the lane, *RT* reaction time, *SM* signaling mistakes, *ES* engine stalls.

### Correlation between visual and driving performance

**Figure 4:**
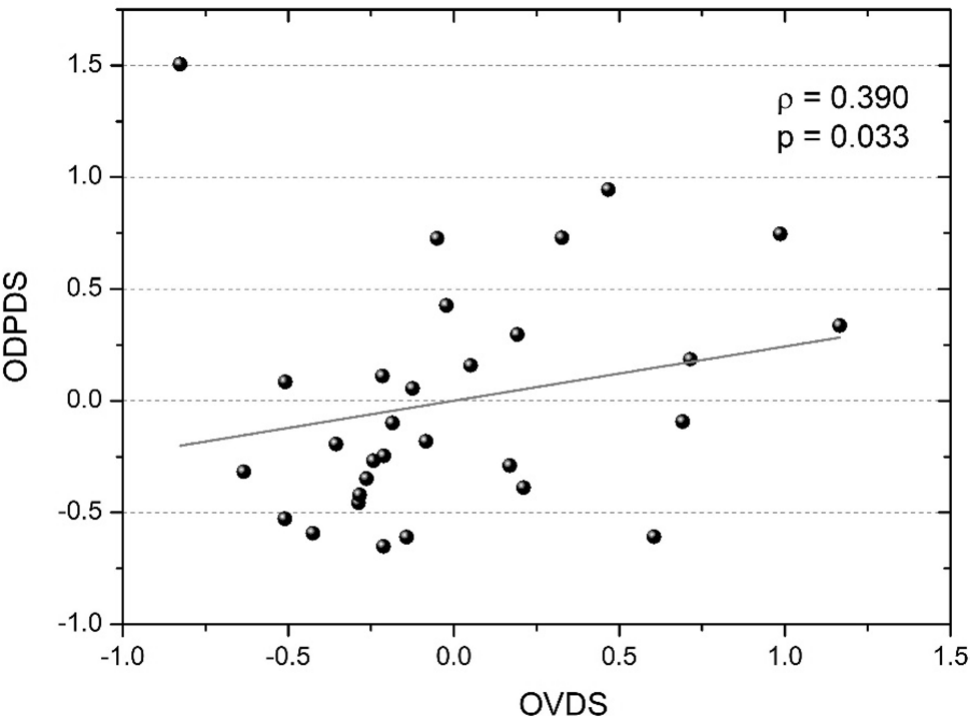
The overall visual deterioration score (OVDS) as a function of the overall driving performance deterioration score (ODPDS).

Figure 4 shows the relationship calculated between the overall visual deterioration (OVDS) and the overall driving performance deterioration (ODPDS). A positive statistically significant correlation (ρ = 0.390, p = 0.033) was found between OVDS and ODPDS, revealing that the greater the deterioration in this overall visual function, the greater the impairment in the driving performance measured. For this reason, degradation in the vergence system and stereoacuity at near and distance, as well as visual acuity, correlate with a consequent deterioration in driving performance. The correlation between OVDS and ODPDS would be greater and more significant if we deleted the outlier (OVDS = − 0.83, ODPDS = 1.50) from the statistical analysis (ρ = 0.528, p = 0.003).

## Discussion

This study provides an overview of the influence of alcohol consumption (BrAC of 0.40 mg/l) on binocular visual performance and simulated driving in healthy young subjects with normal binocular vision, corresponding to the most typical visual condition. We assessed binocular visual performance by means of parameters such as visual acuity, stereoacuity, and near and distance vergence function (phorias, NFV, PFV, AC/A ratio, vergence facility, Sheard’s and Percival’s criteria). Simulated driving performance was characterized by driving parameters for the three road environments: mean speed, SDLP, distance traveled invading the opposite lane, distance traveled invading on the shoulder, total distance traveled outside the lane in the two-lane mountain road, reaction time, signaling mistakes, engine stalls, and number of collisions. All the binocular visual and vergence functions and simulated driving parameters showed a statistically significant impairment following alcohol intake, except near horizontal phoria, near and distance vertical phoria, near Sheard’s and Percival’s criteria and signaling mistakes. In fact, for some parameters measured, distance vergence function showed a greater deterioration after alcohol consumption than near vergence function, except for positive and negative fusional vergences. To the best of our knowledge, there are no previous findings showing correlations between binocular visual performance impairment, specifically vergence function parameters, and deterioration in simulated driving performance due to alcohol consumption. In addition, studying the influence of alcohol consumption on binocular vision and driving performance at the legal BrAC limit of 40 mg/l (BAC of 0.8 g/l or 0.08%) is of special interest for road traffic applications. For the driving task, the present study shows impairment for a BrAC of 0.40 mg/l. Even though the most common legal limit of BrAC worldwide is 0.25 mg/l (BAC of 0.5 g/l or 0.05%), for example in Spain and 51 other countries^1^, we chose a BrAC of 0.40 mg/l because it is still the legal limit for driving in 45 countries around the world, including the UK and the USA^1,58^. Therefore, another aim of this study was to quantify and simulate binocular visual impairment during a highly demanding visual task such as driving. The study evidenced a high deterioration in simulated driving and binocular visual at a BrAC of 0.40 mg/l, as it caused adverse effects in the visual system and diminished driving safety.

Firstly, we analyzed binocular vision function by measuring visual acuity under photopic conditions. Alcohol consumption caused a statistically significant deterioration of 0.26 (decimal notation). As reported by Lee et al.^59^, alcohol consumption is associated with a deterioration of visual acuity. Some investigations have shown that alcohol consumption also negatively affects other visual functions. Castro et al.^18,19^ found a deterioration in visual discrimination capacity in night-vision conditions after drinking red wine. They also found an increase, on average, in pupil diameter in dim surrounding conditions after consuming red wine (mean BrAC of 0.32 ± 0.14 mg/l; mean increase in pupil diameter of 0.4 mm) and a deterioration of retinal image quality. Other authors have also reported an increase in intraocular straylight after consuming alcohol and a deterioration in binocular contrast sensitivity function^34^. The present study corroborates an increase of mean pupil diameter (0.7 mm) induced by a BrAC of 0.40 mg/l which could contribute to poorer retinal image quality^18^. In addition, we found that near and distance stereoacuity also deteriorated following alcohol consumption. The impairment was higher in distance stereoacuity (94 arc sec) compared to near stereoacuity (16 arc sec). Wegner and Fahle^60^ observed a significant deterioration (93 arc sec) in distance stereoacuity after consuming alcohol (a BAC from 0.8 to 1.3 g/l). Watten and Lie^61^ also found near stereoacuity deterioration (32 arc sec) with higher alcohol consumption (BrAC of 0.50 mg/l) than in our study. Stereopsis represents the highest level of binocular vision and is necessary for effective efficient binocular vision quality. A greater impairment in distance stereoacuity implies a deterioration in depth perception and therefore the visual system has a lesser capacity to estimate relative distances. Alcohol consumption had a negative effect on stereoacuity and, consequently, binocular vision performance.

Secondly, to maintain normal and efficient binocular vision and avoid diplopia, sufficient fusional reserves are required to compensate horizontal phoria. Otherwise, subjects may experience visual discomfort, causing tropia, amblyopia, and strabismus. In the present study, results showed that fusional reserves decreased significantly with alcohol consumption, compromising normal binocular vision. In fact, the mean horizontal phoria at distance in the baseline session was − 0.1 ∆ exophoria (normal healthy population) and after consumption was 2.9 ∆ esophoria, which represents an average that falls outside normal values for a healthy population^56,57^. So, a mean increase of 3 ∆ in esophoria was observed with a BrAC of 0.40 mg/l. This is in line with others studies which reported a change towards a more esophoric distance horizontal phoria with alcohol consumption^25,26,62^. Hogan and Lindfield^25^ suggested that ethanol has a toxic effect on the sixth cranial nerve, causing an increase in distance esophoria and subsequently convergent strabismus. Owens and Leibowitz^63^ assumed that alcohol induces esophoria for distance vision, thus causing a regression of vergence toward a tonic position. A possible explication could be that alcohol consumption changes the visual axes towards a convergent position and produces a possible decline in the lateral recti’s ability to contract/relax at distance, thereby impacting on neuromuscular control. On the other hand, for near horizontal phoria, we observed a mean increment (but not significant) of exophoria (1.6 ∆) for a BrAC of 0.40 mg/l. This result is in line with Hogan and Linfield^25^, who found an increase in exophoria for near horizontal phoria of 2 ∆. Munsamy et al.^26^ also observed a significant increase in exophoria of 1 ∆ for a BrAC of 0.50 mg/l. So, for near horizontal phoria, alcohol has a negative effect, deflecting the visual axes towards a divergent position, possibly due to an inhibition of the medial recti muscle. Wilson and Mitchell^64^ associated the increase of exophoria at near with a reduction in muscle tonus and reported that it increased with the amount of alcohol consumed.

We did not find any significant differences for vertical phoria at near and distance, suggesting that alcohol consumption at a BrAC of 0.40 mg/l does not affect vertical muscles and visual axes^25,26,64^. Munsamy et al.^26^ found significant impairments in horizontal positive and negative fusional vergences after alcohol consumption (BrAC of 0.25 mg/l and 0.50 mg/l). In our study, we confirmed that all the mean horizontal positive and negative fusional vergences decreased significantly at near and at distance after consuming alcohol (BrAC of 0.40 mg/l). Moreover, in the break and recovery points, a BrAC of 0.40 mg/l induced a statistically significant impairment of fusional reserves, causing a significant impairment in the Percival’s and Sheard’s criteria at distance. Furthermore, the mean Sheard’s criterion at distance was positive (1.3 ∆), which would cause significant discomfort in binocular viewing conditions (this value is indicative of the need for a prism prescription due to weak binocular vision). Hence, Sheard’s criterion might be of interest when assessing binocular vision in driver eyesight. Hence, the legal alcohol content of 0.40 mg/l, still in force in many countries, weakened binocular fusional reserves, leading to the risk of misaligned visual axes and decompensated phoria. This could induce a collapse in distance phoria from a latent to a manifest deviation resulting in a deterioration of normal binocular vision (strabismus and/or diplopia)^21,25^. Miller et al.^65^ stipulated that the general effect of alcohol is to produce a decrease in vergence range manifesting as changes in both fusional and accommodative vergence. In fact, in our study, the mean significant decrease and deterioration were apparent in the calculated AC/A ratio (1.7 ∆) and in the gradient AC/A ratio (1.8 ∆). Cohen and Alpern^66^ also found a decrease in the AC/A ratio for moderate alcohol intake, whereas Hogan and Linfield^25^ did not find a significant change in the AC/A ratio for a low alcohol intake. Rosenfield et al.^37^ suggested that the oculomotor crosslinks are innervated by the combined output of the fast and slow controllers and the accommodative convergence by fast and slow blur-driven accommodation, which implies that alcohol consumption could have a toxic effect. The mean vergence facility at near was impaired at a BrAC of 0.40 mg/l. In fact, for this parameter, alcohol consumption induced a statistically significant deterioration of 5.5 cpm, which agrees with our results which indicate weakness of binocular visual performance.

A change from natural to less natural conditions due to alcohol consumption led to a decrease in vergence facility^45^. Vergence facility represents a clinically useful parameter for the diagnosis of binocular vision abnormalities, and is closer to the dynamic character of real-life situations^43^. However, no studies have been published about the influence of alcohol consumption and the corresponding effect on vergence facility. We measured vergence facility following a moderate alcohol intake, which highlighted the deterioration of the ability of the fusional vergence system to change vergence demands. A deterioration in this parameter due to alcohol intake could cause binocular-vision difficulties in real situation, like driving, because our visual system demands constant changes between near and far distances.

Thirdly, another goal of the present study was to use a driving simulator to assess how alcohol consumption deteriorates binocular visual performance and subsequently impairs a highly visual task such as driving. In the present study, the mean speed was higher in the three road sections (dual carriageway, two-lane mountain road, and inner city) with a BrAC of 0.40 mg/l. The greatest increase in mean speed was recorded for the dual carriageway, exceeding the legal driving limit of 120 km/h. In accordance with other studies, mean speed rises for a legal BAC of 0.5 g/l or 0.8 g/l^4,67,68^. Mean speed SD also increased in the three sections following alcohol consumption, indicating increased difficulty in maintaining a constant velocity.

The SDLP is a valid parameter for measuring how much drivers weave across the road after consuming alcohol^54^. In our study, the SDLP was worse in the dual carriageway and in the two-lane mountain road, which indicates that subjects found it harder to follow road trajectories. Others studies confirmed a higher SDLP with a BAC of 0.5 g/l^55^ and 0.8 g/l^27,68^ (BrACs of 0.25 and 0.40 mg/l, respectively), and for a BrAC above 0.25 mg/l^34^. In the present study, the distance traveled invading the opposite lane or the distance traveled invading the shoulder significantly increased with a BrAC of 0.40 mg/l. This was congruent with Allen et al.^8,9^ who also found that lane deviation increases at a BAC level of 0.55 and 1.1 g/l (BrACs of 0.28 and 0.55 mg/l, respectively), which was explained through lower driver control parameters. Moreover, our results confirmed that after consuming alcohol, the greater the inherent difficulty in following the road (the two-lane mountain road with respect to the dual carriageway), the greater the likelihood of crossing lane boundaries, that is, invading the opposite lane or the shoulder. These parameters confirmed that drivers found it harder to control the position of the car in simulated driving under the influence of alcohol. 

Another important factor to evaluate during driving is the reaction time^4^, defined as the capacity of the psychomotor reflex to respond in time with respect to a driving situation. In our study, the mean braking reaction times increased significantly between baseline and after consuming alcohol. This indicates that the behavioral reflexes declined with the influence of alcohol. Others studies investigated this parameter with different levels of alcohol (BAC levels of 0.35, 0.4, 0.5, 0.6, 0.8, and 1.1 g/l), and they all reported an increase in reaction time^2,9,69,70^. At a BAC level of 0.8 g/l (equivalent to a BrAC of 0.40 mg/l), Jongen et al.^70^ obtained a significant increase of 0.209 s in the psychomotor reaction time. They suggested that one of the most common effects of alcohol relevant to potential driving impairment is sedation or drowsiness associated with slower responses and attention deficits^70^. Banks et al.^69^ also established that an increase in electroencephalogram-measured somnolence impaired driving simulator performance in parameters such as reaction time and number of collisions. Khan and Timney^20^ found that the decline in driving performance was due to an impairment in a variety of perceptual and motor systems and the failure to process information correctly. Alcohol can also increase the incidence of distraction-related behaviors, impacting simulated driving performance^71^. With this in mind, in our study, the mean collision parameter also increased significantly for the BrAC level of 0.40 mg/l (by a factor of 4.3). In all the simulated driving parameters we measured, a BrAC of 0.40 mg/l had a considerable effect on the subjects’ behavior, affecting their capacity to drive safely.

Fourthly, we found a correlation between visual deterioration (including fusional reserves, stereoacuity and visual acuity) and simulated driving impairment, revealing that these visual parameters influence driving performance. In this research, the overall visual performance is mainly constituted by fusional vergences and stereoacuity, highlighting the importance of these binocular functions on the simulated driving performance. Stereopsis is considered the most advanced state of binocular vision, contributing to seeing depth (three-dimensional perception)^72^. Although stereopsis is usually characterized by maximum disparity^72^ and stereoacuity, the latter is the main visual function used in clinical practice, and it is sensitive to ocular changes, influencing binocular visual performance^73^. Therefore, we consider that far stereoacuity could be considered in visual examinations for driving licenses given its widespread use to characterize binocular vision^10^, since it could play an essential role in driving safely. It should be taken into consideration that some monocular cues contribute to obtaining information on relative distances and depth in the perceived scene, which seem to be sufficient to meet driving standards and drive safely. In fact, in the absence of binocular vision and stereopsis, driving under monocular viewing conditions is allowed in most countries^14^. However, it has been demonstrated that an induced deterioration of binocular vision and monocular viewing conditions negatively influences simulated driving^74^ and monocularity significantly deteriorates driving performance under more demanding conditions, such as car racing^75^. We centered our investigation on driving under normal binocular viewing conditions, the most typical natural conditions, although it would be of interest in future work to study the implications of monocularity on driving performance in challenging conditions like those studied here, i.e., after alcohol intake. Casares-López et al.^34^ demonstrated that other visual parameters such as contrast sensitivity and retinal straylight have an important impact on simulated driving after consuming alcohol^34^. However, they stated that the contribution of these two visual variables was limited (16.1%), indicating that other visual functions or psychomotor aspects may also influence driving ability after alcohol consumption. This limitation is also present in our study, although we have more completely analyzed binocular vision through different visual functions, and maintained a stable alcohol content of 0.40 mg/l. However, in further investigation it would be interesting to consider the effects of both visual and psychomotor aspects on driving under the influence of alcohol.

Although subjects were instructed to drive normally and respect the traffic rules just as they would in real life, the study may be limited by the fact that subjects do not interpret risk in the experimental task with the same realism as they do in real-world driving. However, we used a driving simulator because it represents an efficient, applicable, and safe device to assess driving performance^8,9,32,50^ in both natural and challenging conditions (such as under the influence alcohol).

Our findings could help provide a better understanding and quantification of the effect of alcohol consumption on binocular visual performance (focusing particularly on the vergence system and stereopsis) with respect to an important everyday task such as driving. This study represents an overview of the influence of alcohol on binocular vision and simulated driving. In fact, it is very remarkable that even a moderate dose of alcohol such as the one employed in this work, which is the legal limit for driving in 45 countries around the world, significantly diminishes binocular vision and driving capacity. This could tragically increase the incidence of traffic accidents. Visual acuity (with a binocular vision of at least 0.5) is commonly used as a visual parameter during tests to obtain a driving license in most countries around the world^14,76^. In our study, for a BrAC of 0.40 mg/l, the mean binocular visual acuity remained better than 0.5, measured at 1.09. Even with this good level of binocular visual acuity, our results showed a significant impairment in binocular visual performance and driving ability. Therefore, other binocular vision parameters should also be considered for driving license eye tests, such as stereoacuity and a complete vergence exam, to ensure individuals have a safe level of binocular vision.

Further investigation focused on driving performance in people with monocular vision would be of interest. In fact, this particular driving situation could be compared to the normal binocular scenario under various experimental conditions, such as under the influence of alcohol. It could also be of interest to study how motor function and visual deterioration impair driving performance.

## Conclusion

The present study analyzes a complete overview involving the vergence system, binocular visual function (by means of the VA and stereoacuity), and driving parameters (mean speed, SDLP, distance traveled invading the opposite lane, reaction time, number of collisions, etc.) in a baseline session and after alcohol consumption. In the binocular vision, we found strong deteriorations at a BrAC of 0.40 mg/l in stereoacuity, fusional reserves (PFV, NFV, and vergence facility), AC/A, Sheard’s criterion at far, and horizontal phoria at distance causing a decrease in visual binocular performance. We also observed a positive correlation between deterioration of binocular vision and impairment on simulated driving performance. In fact, this is the first study that correlates binocular visual deterioration including fusional reserves with simulated driving performance after consuming a moderate amount of alcohol. Our findings suggest that other binocular vision parameters could be considered for driving license tests such as stereoacuity and a complete vergence exam to ensure a safe binocular vision performance. Therefore, the present research shows the importance of a complete binocular vision exam in drivers to provide a more thorough assessment of the visual system. This study also emphasizes the risk reduced vision and driving performance with moderate alcohol consumption. The use of awareness campaigns could help communicate the hazards of alcohol consumption with respect to road and driver safety due to the difficulty of driving when binocular vision is impaired by alcohol intake.

## Data Availability

The datasheet is available in Zenodo, the open-access repository developed under the European OpenAIRE program and operated by CERN. 10.5281/zenodo.4516681.
